# Expanding deep phenotypic spectrum associated with atypical pathogenic structural variations overlapping 15q11–q13 imprinting region

**DOI:** 10.1002/brb3.3437

**Published:** 2024-04-14

**Authors:** Rabeya Akter Mim, Anjana Soorajkumar, Noor Kosaji, Muhammad Mizanur Rahman, Shaoli Sarker, Noushad Karuvantevida, Tamannyat Binte Eshaque, Md Atikur Rahaman, Amirul Islam, Mohammod Shah Jahan Chowdhury, Nusrat Shams, K. M. Furkan Uddin, Hosneara Akter, Mohammed Uddin

**Affiliations:** ^1^ Genetics and Genomic Medicine Centre (GGMC) NeuroGen Healthcare Dhaka Bangladesh; ^2^ Center for Applied and Translational Genomics (CATG) Mohammed Bin Rashid University of Medicine and Health Sciences, Dubai Health Dubai UAE; ^3^ Department of Paediatric Neurology Bangabandhu Sheikh Mujib Medical University Dhaka Bangladesh; ^4^ Bangladesh Shishu Hospital and Institute Dhaka Bangladesh; ^5^ GenomeArc Inc. Mississauga Ontario Canada; ^6^ Ministry of Health and Family Welfare Dhaka Bangladesh; ^7^ National Institute of Neuroscience and Hospital Dhaka Bangladesh

**Keywords:** 15q11–q13 duplication syndrome (Dup15q syndrome), Angelman syndrome, chromosome 15q11–q13 region, Prader–Willi syndrome

## Abstract

**Background:**

The 15q11–q13 region is a genetic locus with genes subject to genomic imprinting, significantly influencing neurodevelopment. Genomic imprinting is an epigenetic phenomenon that causes differential gene expression based on the parent of origin. In most diploid organisms, gene expression typically involves an equal contribution from both maternal and paternal alleles, shaping the phenotype. Nevertheless, in mammals, including humans, mice, and marsupials, the functional equivalence of parental alleles is not universally maintained. Notably, during male and female gametogenesis, parental alleles may undergo differential marking or imprinting, thereby modifying gene expression without altering the underlying DNA sequence. Neurodevelopmental disorders, such as Prader–Willi syndrome (PWS) (resulting from the absence of paternally expressed genes in this region), Angelman syndrome (AS) (associated with the absence of the maternally expressed *UBE3A* gene), and 15q11–q13 duplication syndrome (resulting from the two common forms of duplications—either an extra isodicentric 15 chromosome or an interstitial 15 duplication), are the outcomes of genetic variations in this imprinting region.

**Methods:**

Conducted a genomic study to identify the frequency of pathogenic variants impacting the 15q11–q13 region in an ethnically homogenous population from Bangladesh. Screened all known disorders from the DECIPHER database and identified variant enrichment within this cohort. Using the Horizon analysis platform, performed enrichment analysis, requiring at least >60% overlap between a copy number variation and a disorder breakpoint. Deep clinical phenotyping was carried out through multiple examination sessions to evaluate a range of clinical symptoms.

**Results:**

This study included eight individuals with clinically suspected PWS/AS, all previously confirmed through chromosomal microarray analysis, which revealed chromosomal breakpoints within the 15q11–q13 region. Among this cohort, six cases (75%) exhibited variable lengths of deletions, whereas two cases (25%) showed duplications. These included one type 2 duplication, one larger atypical duplication, one shorter type 2 deletion, one larger type 1 deletion, and four cases with atypical deletions. Furthermore, thorough clinical assessments led to the diagnosis of four PWS patients, two AS patients, and two individuals with 15q11–q13 duplication syndrome.

**Conclusion:**

Our deep phenotypic observations identified a spectrum of clinical features that overlap and are unique to PWS, AS, and Dup15q syndromes. Our findings establish genotype–phenotype correlation for patients impacted by variable structural variations within the 15q11–q13 region.

## Graphical abstract

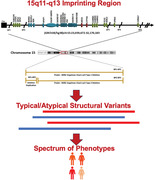

## 1 INTRODUCTION

The chromosomal 15q11–q13 region is characterized by its complex structure, which contains several genes that are subject to genomic imprinting, a phenomenon where genes are preferentially expressed from one parental allele. Structural variations (SV) involving deletions or duplications in this region are associated with three distinct neurodevelopmental disorders (NDDs): Prader–Willi syndrome (PWS), Angelman syndrome (AS), and 15q11–q13 duplication syndrome (Dup15q syndrome). In rare developmental and NDDs, disease‐associated genetic variants lead to a manifest spectrum of phenotypes and often overlap with other syndromes or disorders (Birnbaum et al., 2022; Safizadeh Shabestari et al., 2022; Woodbury‐Smith et al., 2017a, 2017b). The clinical presentation of disorders at 15q11–q13 can vary widely and is influenced by genetic and environmental factors. Each of these disorders is caused by either the loss of function or overexpression of at least one imprinted gene, and their frequency of occurrence is approximately 1 in 15,000–30,000 live births (Kalsner & Chamberlain, 2015a). Unfortunately, it is not known how fragile 15q11–q13 critical regions are in ethnically diverse populations across the world.

In the 15q11–q13 breakpoints, PWS, AS, and Dup 15q syndrome result in different genotypes due to the imprinted nature of the locus. PWS (OMIM #176270) is a rare genetic disorder with an estimated incidence of 1:10,000–1:30,000 live births (Cassidy et al., 2012). PWS is the result of deficiencies in almost 20 paternally expressed genes and is classified into 3 molecular types, including a paternal 15q11–q13 deletion (70% of cases), maternal uniparental disomy 15 (UPD15), in which both chromosomes 15 are inherited from the mother (25%), and an imprinting center (IC) defect (4%) (Bittel & Butler, 2005; Grootjen et al., 2022; Kim et al., 2012). Microdeletions or epimutations of the IC, which control the expression status of selected imprinted genes on chromosome 15, can cause PWS on the paternal allele. SVs often harbor genes that are critical for development (Uddin et al., 2014), and *SNRPN* resides within the PWS critical region, 15q11–q13 (Lee & Wevrick, 2000; Meguro et al., 2001; Nicholls, 1999), which is considered a prime candidate gene. The clinical phenotypes of PWS are highly variable, and the syndrome shows changing clinical features during a patient's life. Most cases of PWS are sporadic, with an approximately equal incidence among ethnic groups and sexes. Common features include infantile hypotonia, a poor suck reflex with feeding difficulties, short stature with small hands and feet, hypogonadism secondary to hormone deficiencies, behavior problems, and hyperphagia (Milner et al., 2005).

On the other hand, Angelman syndrome (OMIM #105830) is a rare NDD characterized by the lack of expression of the maternal ubiquitin–protein ligase E3A (*UBE3A*) gene in the brain (Kishino et al., 1997; Matsuura et al., 1997). Four known etiologies of AS lead to the silencing of the *UBE3A* gene: deletion in chromosome 15q11–q13 (70% of cases), paternal UPD (2% of cases), imprinting defect (3% of cases), and point mutation (10% of cases) (Beygo et al., 2019; Buiting et al., 2016). However, approximately 10%–15% of clinically diagnosed AS patients have an unknown genetic cause (AS‐like). The prevalence of AS is generally estimated to be approximately 1:15,000 births, although the true prevalence is not well characterized. The clinical phenotype of AS is characterized by seizures, severe developmental delay, absent or severely limited speech, gait ataxia and/or tremulousness of the limbs, and a unique behavior with a happy demeanor (Beygo et al., 2019; Bird et al., 2014; Maranga et al., 2020; Smith, 2001; Williams et al., 2001). The consensus criteria for the clinical diagnosis of AS were proposed by Williams et al. (2006), which included a list of consistent, frequent, and associated features. These consensus criteria are typically used as a basis for a clinical diagnosis, which is then confirmed through genetic testing.

Lastly, Dup15q syndrome is a rare genetic disorder that affects approximately 1 in 30,000 births and expresses double dosages of the imprinted genes either in the maternal locus or the paternal locus, depending on the methylation pattern of the duplicated region. This condition is characterized by the presence of at least one extra copy in the Prader–Willi/Angelman Critical Region of the referred 15q11.2–q13.1 chromosome (Mao et al., 2000). In 80% of cases, the extra genetic material is due to the presence of extrachromosomal information, leading to a supernumerary chromosome, a condition known as isodicentric 15q11.2–q13.1 duplication (Idic15q). In the remaining 20% of cases, additional genetic material is duplicated within the chromosome itself, resulting in an interstitial duplication of the 15q11.2–q13.1 chromosome (Dup15q) (Wang et al., 2015). Isodicentric duplications are always considered to be de novo, whereas maternal interstitial duplications are de novo in 85% of cases and maternally inherited in 15% of the remaining cases (Cassidy et al., 2000; Finucane et al., 1993). Individuals with Dup15q syndrome commonly present with hypotonia, developmental delay ranging from mild to severe, risk of epilepsy, and varying severities of ASDs (Mao et al., 2000; Urraca et al., 2013).

Our study aims to identify clinical heterogeneity among patients impacted by SVs within the 15q11–q13 region. Whole genome microarray was performed to investigate the frequency of associated deletion and duplication mutations, and comprehensive phenotypic profiling was analyzed to highlight patterns and distributions across different disease cases. Thorough phenotypic clinical observation can help clarify the previously ambiguous landscape of overlapping clinical features from neurodevelopmental and neurometabolic disorders.

## 2 MATERIALS AND METHODS

### 2.1 Study subjects and deep clinical assessment

The study was conducted in accordance with ethical guidelines and received approval from the Institutional Review Board of Holy Family Red Crescent Medical College and Hospital in Dhaka, Bangladesh (IERC/20/Res/Nov/2017/30). The study cohort (*N* = 260) was derived from the NeuroGen Healthcare database located in Dhaka, Bangladesh. Referrals of patients in this cohort came from various child neurologists at different tertiary‐level clinics and hospitals, aiming to investigate genetic aspects of NDDs. During clinical visits, a team of experienced pediatric neurologists performed thorough neurological and neurodevelopmental assessments to identify potential individuals and establish clinical diagnoses based on the observed features.

A retrospective cohort study was undertaken, with data pertaining to eight patients extracted from a larger study cohort (*N* = 260) that previously underwent whole‐genome microarray analysis. The comprehensive genotype and phenotype‐based analysis of the 260 participants was conducted by Akter et al. (2023), where various known syndromes as well as novel candidate copy number variation (CNVs)/genes were identified. The study revealed the most frequent CNVs associated with PW/AS, including two duplications associated with Dup15q syndrome and three deletions associated with PW/AS. Subsequently, an additional three PWS patients were identified within the 260 cohort, bringing the total number of patients included in this study for a detailed genotype–phenotype association to eight.

Prior to enrollment, individuals with suspected PWS/AS were invited to participate and provided written consent forms, which were approved by their parents or legal guardians. A comprehensive physical examination, including morphometric assessments, was performed to gather detailed data on the patient's physical characteristics. Demographic and clinical information for the patients was obtained through formal parental interviews conducted during the sample collection at the clinic. This information encompassed baseline demographics, such as age, gender, and birth history, as well as clinical details, including developmental histories, medical histories, family histories, infection histories, dietary details, and behavioral issues. All patients underwent in‐depth clinical phenotyping, which involved multiple examination sessions to evaluate a wide range of clinical symptoms.

### 2.2 Genomic DNA extraction

Peripheral blood sample (10 mL) was collected from patients in an EDTA vacutainer tube. Genomic DNA was extracted using the ReliaPrep Blood gDNA isolation kit (Promega), following the protocols detailed in the kit. The quality and quantity of DNA were determined using NanoPhotometer C40 (Implen) and resolved on 0.8% agarose gel.

### 2.3 Whole‐genome microarray

We conducted genome‐wide microarray to identify chromosomal abnormalities, such as deletions, duplications, translocations, and rearrangements. Changes in fluorescence intensity between the test specimen and the controls were investigated using array‐comparative genomic hybridization chip technology in the Agilent system. Digestion, ligation, PCR, labeling, hybridization, and scanning were all performed following standard protocols. This microarray uses 33,000 probes spread across the genome to detect 372 genetic abnormalities (including >60 loci in the DECIPHER database reported for NDDs) and targets 41 subtelomeric regions that are vulnerable to chromosomal abnormalities. We rigorously applied multiple algorithmic techniques (MATLAB and Java) and manually curated the data to pinpoint genomic variation based on the normalized log2 intensities of the probes (see the Supporting Information section for details). A circular binary segmentation algorithm was used on the normalized –log2 values to detect CNVs. Horizon platform from GenomeArc Inc. was used for clinical annotations of the SVs and classifies pathogenicity following American College of Medical Genetics guidelines. Horizon algorithm was integrated with in‐house control population (9610 samples) to exclude common CNVs and retain only rare CNVs for clinical annotation. We also integrated the Horizon platform with previously published neurodevelopmental cohorts to identify variant frequencies in disease cohorts (Akter et al., 2023; Uddin et al., 2016).

### 2.4 NDD disorder enrichment

We have screened all known disorders from the DECIPHER database and identified variant enrichment within this cohort. CNVs can vary in actual length compared to the breakpoints mentioned in the disorder database. Applying the Horizon analysis platform, we have conducted enrichment analysis by reciprocal mapping the CNV breakpoints, where at least >60% of the variant must overlap between a CNV and a disorder breakpoint. Our analysis demonstrates that the 15q11–q13 imprinting region shows the most enriched number of NDD cases within the cohort. We have proceeded with all related downstream analyses (variant validation, appointment for deep phenotype information) based on the cases that are within the 15q11–q13 imprinting region.

### 2.5 Droplet digital PCR

Copy number assays were performed using the Droplet Digital PCR (ddPCR) system (Bio‐Rad Laboratories, Inc.). The GeneAssist Copy Number Assay Workflow Builder (Thermo Fischer) was used to design TaqMan assays on Chr15, SNHG14 (Hs05375107_cn) with FAM dye. TaqMan Copy Number Reference Assay, human, RNase P with VIC dye was used as a reference assay. A total of 22 μL reaction mix was prepared, containing 3.5 μL of template DNA (20 ng/μL) without restriction digestion, 10 μL of 2× ddPCR supermix for probes (no UDP) (Bio‐Rad Laboratories Inc.), 1 μL each 20× TaqMan target probe (FAM) and 20× TaqMan reference probe (VIC) (Applied Biosystems), and 6.5 μL of RNase‐/DNase‐free water (see the Supporting Information section for details). All reactions were prepared in triplicates with one negative control. The reaction mixtures were partitioned using the QX200 Droplet Generator and then transferred to a 96‐well plate and amplified using the C1000 Touch thermal cycler, following the manufacturer's protocol. The samples were then read using the QX200 Droplet Reader. Data acquisition and analysis were performed using QuantaSoft Version 1.7.4.0917, and the Poisson algorithm was used to determine the concentrations of the targets as copies/μL.

## 3 RESULTS

### 3.1 Patient's clinical features

The deep phenotypic analysis involved eight patients, aged between 1 and 6.5 years, with a mean age of 3.4 years. During routine general physical examination, these patients exhibited a diverse range of symptoms (Table 1 and Figure S1), including global developmental delay, which was observed in all eight patients (100%). For deep clinical phenotyping, multiple examination session was conducted to evaluate numerous clinical symptoms. Due to certain limitations, psychological assessments for patients suspected of having intellectual disability were not performed. There are limited genomic studies reported from Bangladesh on rare developmental and NDDs (Akter et al., 2021, 2023; Rahman et al., 2019; Uddin et al., 2018). The observed clinical features in eight patients in the Bangladeshi cohort are summarized in Figure S1. More than 50% of the patients showed most of the observed clinical features with 75% (6/8) patients displaying floppiness as a baby and poor socialization, and 62% (5/8) patients displaying hyperactivity, facial dysmorphisms, hypotonia, and pica. Seizures were present in 50% of the patients (4/8), as were rapid weight gain, feeding difficulties, and sleeping problems. Intellectual disability was observed in 25% (2/8) of the patients. Additionally, almost 37% (3/8) of patients displayed genital deformities such as a small penis or labia, nausea, and short stature.

**TABLE 1:** Deep phenotypic characteristics for eight neurodevelopmental disorder patients with atypical and typical pathogenic structural variation (SV) breakpoints within the 15q11–q13 region.

Demographics		HPO code	Patient 1	Patient 2	Patient 3	Patient 4	Patient 5	Patient 6	Patient 7	Patient 8
**Clinical features**	**Age**		3.9	3	1	4	1.9	4.9	6.5	2.5
	**Birth weight (kg)**		2.6	2.4	1.5	3	1.6	1.8	2.7	2.9
	**Gender**		Male	Male	Female	Female	Male	Male	Female	Male
	**Facial dysmorphism**	HP:0000271	No	Yes	Yes	No	Yes	Yes	No	Yes
	**Floppy as a baby**		Yes	Yes	Yes	No	Yes	Yes	No	Yes
	**Colic**	HP:0025429	No	No	Yes	No	No	No	Yes	No
	**Global developmental delay**	HP:0001263	Yes	Yes	Yes	Yes	Yes	Yes	Yes	Yes
	**Intellectual disability**	HP:0001249	No	Yes	No	No	No	Yes	Yes	No
	**Hypotonia**	HP:0001252	No	Yes	Yes	No	Yes	Yes	No	Yes
	**Seizure**	HP:0001250	No	Yes	No	No	No	Yes	Yes	Yes
	**Feeding difficulties**	HP:0011968	No	Yes	Yes	No	Yes	Yes	No	No
	**Sleeping problems**	HP:0002360	No	Yes (mild sleep apnea)	No	No	Yes (excessive sleepiness)	Yes (sleepiness in daytime)	No	Yes (decreased sleep need)
	**Rapid weight gain**	HP:0004324	No	Yes	No	Yes	Yes	Yes	No	No
	**Small penis**	HP:0000054, HP:0000059	No	Yes	No data	No	Yes	Yes	No	No
	**Short stature**	HP:0004322	No	No	No	Yes	Yes	Yes	No	No
	**Narrow hand**	HP:0004283	No	No	Yes	No	Yes	Yes	No	No
	**Fair skin**	HP:0007513	No	Yes	No	No	No	Yes	Yes	Yes
	**Thick viscous saliva**		Yes	No	No	No	Yes	No	No	No
	**Rarely vomits**	HP:0002017	No	Yes	No	Yes	Yes	No	No	No
**Behavioural abnormalities**	**Poor socialization/Cognition**	HP:0000735, HP:0100543	Yes	Yes	No	Yes	No	Yes	Yes	Yes
	**Hyperactivity & aggressiveness**	HP:0000752, HP:0000718	No	Yes	Yes	Yes	No	Yes	Yes	No
	**Stereotypic behavior/Autistic features**	HP:0000733, HP:0000729	Yes	No	No	Yes	No	No	No	No
	**Hyperphagia**	HP:0002591	No	Yes	No	No	No	Yes	No	No
	**Try to eat nonedible things**	HP:0011856	No	Yes	Yes	No	Yes	Yes	Yes	No
	**Skin picking**	HP:0012166	No	No	Yes	No	No	No	No	No
	**High pain tolerance**	HP:0007328	Yes	No	Yes	No	No	No	No	No
	**Inattention & impulsivity**	HP:0000736, HP:0100710	No	No	No	No	No	No	Yes	No
**Molecular genetics**	**Deletion/Duplication size (bp)**	‐	4845,577	5273,996	5959,227	7568,766	6160,123	5536,674	4924,147	5527,661
	**Genomic coordinates**	‐	Chr15:	Chr15:	Chr15:	Chr15:	Chr15:	Chr15:	Chr15:	Chr15:
	**Genomic coordinates Disease diagnosed**	‐	23,443,796–28,289,373	23,040,386–28,314,382	22,777,708–28,736,935	20,802,382–28,371,148	22,600,362–28,760,485	22,777,709–28,314,382	23,370,969–28,295,115	22,767,455–28,295,115
		‐	15q duplication	Prader–Willi	Prader–Willi	15q duplication	Prader–Willi	Prader–Willi	Angelman	Angelman

### 3.2 Identification, quantification, and validation of chromosomal abnormalities

**FIGURE 1:**
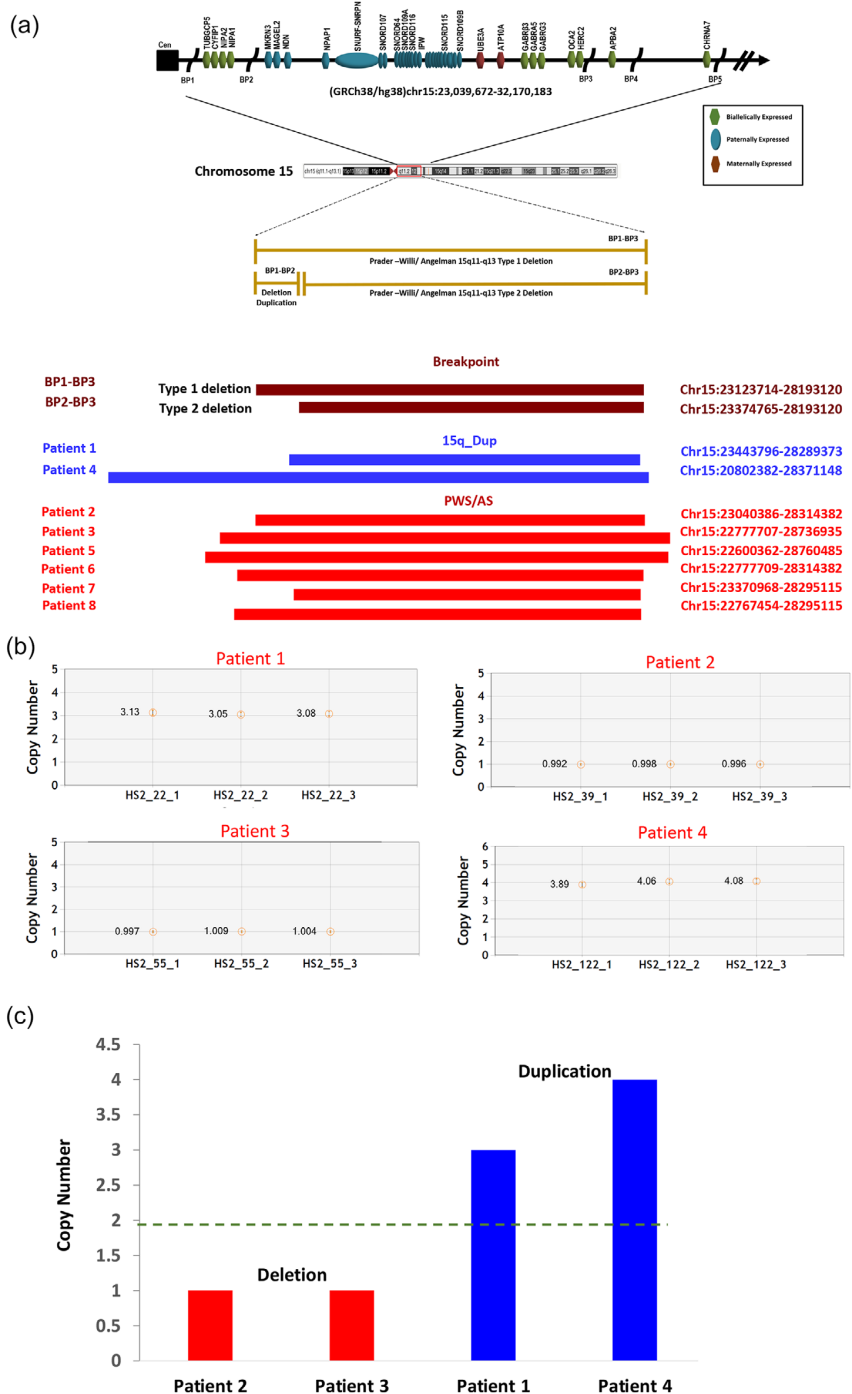
Comprehensive genetic and clinical analysis of eight patients. (a) Illustration of the detected copy number variations (CNVs) in eight patients using chromosomal microarray. Above, the genes and previously reported breakpoints in the 15q11–q13 region are shown in their relative genomic positions. Below are shown BP1–BP3 and the observed deletions and duplications in all the patients. The blue and red bars represent the gross duplications and deletions associated with Prader–Willi syndrome (PWS)/Angelman syndrome (AS)/15q_duplication syndrome, whereas the brown bars indicate the breakpoints. The UCSC genome browser was used to map the estimated base positions for the deleted or duplicated regions to human genome assembly hg38. Type 1 deletion between BP1–BP3 (GRCh38 chr15:23123714‐28193120) and type 2 deletion between BP2 and BP3 (GRCh38 chr15:23374765‐28193120). BP1–BP3 are breakpoints 1–3, respectively. (b) Copy number estimation using digital droplet PCR (ddPCR). The *y*‐axis represents the absolute copy number estimation for each of the patient samples. Each patient experiment has three replicates that show consistent CNV counts. (c) Bar plot showing detected CNVs by ddPCR. The red and blue bars represent the deletions and duplications, respectively.

Upon further analysis of the patients using chromosomal microarray, it was revealed that all eight patients exhibited chromosomal abnormalities in the 15q11.2–q13.1 region of chromosome 15. Remarkably, each patient presented with CNVs within this region, which were either deletions or duplications of varying lengths. The estimated base positions for the deleted or duplicated regions were mapped to human genome assembly hg38 using the UCSC genome browser. Figure 1a illustrates the deletions and duplications observed in this study (Supporting Information and Table 1). For the analysis, we utilized a threshold of LRR (log *R* ratio) ← 0.2 for deletion and >0.2 for duplication. Specifically, six of the observed CNVs were deletions (CNVs Patients 2, 3, and 5–8), whereas two were duplications (CNVs Patients 1 and 4). Notably, Patient 1 exhibited a large 4.8 megabase duplication that spans the BP1–BP3 locus, whereas Patient 7 displayed a 4.9 megabase deletion categorized as a type 2 deletion and spanning the BP2–BP3 region. Intriguingly, the remaining cases demonstrated rare and larger deletions or duplications that extended beyond the BP1–BP3 locus.

To establish the veracity of the CNV data acquired through microarray analysis, we executed further validation procedures utilizing ddPCR. We arbitrarily selected four patient samples (Patients 1–4) and performed triplicates of each sample (Figure 1b). We used *SNHG14* (Hs05375107_cn) as a reference to calculate gene copy numbers, which are known to have two copies. Our results, illustrated in Figure 1b,c, revealed that Patients 2 and 3 displayed an average deletion of 1, whereas Patients 1 and 4 showed duplications of 2 and 4, respectively. Significantly, all four samples are concordant with microarray CNVs, and each test was repeated with three replicates (Figure 1b,c and Table S2).

### 3.3 Genotypic and phenotypic correlations of cases with 15q region mutation

In our study, we have identified different genomic alterations, encompassing eight distinct lengths of deletion and duplication in a patient cohort, leading to disruptions in both imprinted and non‐imprinted genes. Focusing on chromosome 15q11–q13, which harbors numerous genes and transcripts, our analysis revealed 15 imprinted genes (Table S3), including *SNRPN*, *SNURF*, and multiple copies of C/D box snoRNAs involved in RNA processing. These transcripts are encoded by an extended length of the *SNRPN*–*SNURF* gene complex. Among the paternally expressed imprinted genes in this region are *MKRN3*, *MAGEL2*, *NPAP1*, and *NDN*, contributing to neural development, brain function, and aspects of physical, mental, and behavioral changes. Conversely, maternally expressed genes *UBE3A* and *ATP10A* are associated with AS. Noteworthy genes such as GABA receptor subunits and *HERC2*, implicated in neurotransmission and intellectual disability, further add complexity to this genomic region (Butler, 2017).

Within the 15q11–q13 cohort, PWS patients exhibited similar deletion lengths, impacting 15 imprinted genes and approximately 17 non‐imprinted genes, resulting in comparable phenotypes. In contrast, patients with duplication 15q syndrome (Patients 1 and 4) displayed varying duplication lengths, with Patient 4 presenting 24 additional non‐imprinted genes. Phenotypic analysis revealed more severe clinical outcomes in Patient 4, including short stature, rapid weight gain, vomiting tendency, poor socialization, and hyperactivity. Further investigation identified non‐imprinted genes like *NIPA1*, *NIPA2*, *CYFIP1*, and *TUBGCP5* in Patient 4, linked to brain and muscle development, and uniquely associated with attention‐deficit hyperactivity disorder (Davis et al., 2019). Patients 7 and 8, identified as AS cases with deletions of 4.9 and 5.5 megabases, respectively, demonstrated varying degrees of phenotypic severity. Patient 8 exhibited more pronounced features, such as hypotonia, colic, sleeping difficulties, floppiness, and facial dysmorphisms, possibly attributed to the absence of −7 additional non‐imprinted genes in the deletion region. Despite nearly identical deletion sizes in PWS cases, the observed phenotypic variations underscore the intricate interplay of genomic alterations within this region.

### 3.4 Comprehensive clinical profiling of patients with PWS, AS, and 15q11–q13 duplication syndrome

**FIGURE 2:**
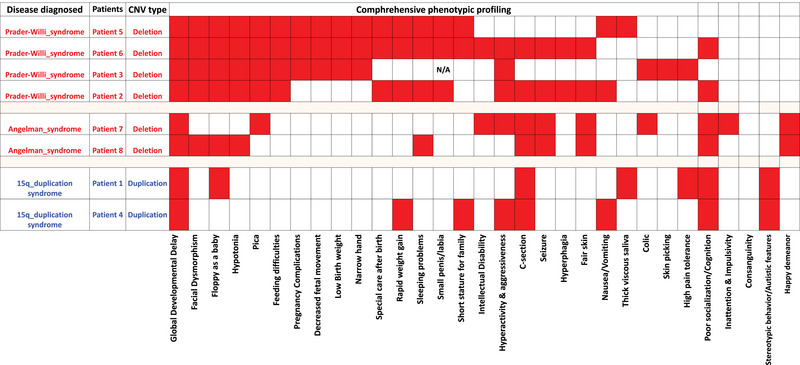
Deep clinical phenotypic profiling, including diagnosed disease, for all eight patients.

Following the detection of deletions and duplications in the 15q11–q13 region, an extensive clinical evaluation was conducted to examine the phenotypic sequelae of all eight patients. Due to certain limitations, we were unable to perform MS‐MLPA to ascertain the methylation status and parental origin of the individuals. Considering both their clinical features and the nature of the mutations, the clinician diagnosed four cases with PWS, two cases with AS, and the remaining two cases with 15q duplication syndrome. The genetic identification and phenotypic profiling of the patients are depicted in Figure 2 and Table 1. Significantly, we found all the cases as pathogenic.

All four cases of PWS demonstrated the incidence of global developmental delay, facial dysmorphism, floppy baby syndrome, hypotonia, and pica, along with feeding difficulties. PWS patients were observed to experience feeding difficulties. Notably, feeding challenges were predominantly observed during early infancy, typically between 0 and 30 days. In later stages of development, 50% (2 of 4) of the cases (specifically noted in Patients 2 and 6, with onset around 2.5 and 3.7 years, respectively) exhibited early‐onset hyperphagia. Furthermore, all of them demonstrated a tendency to consume nonedible items. In addition, 75% of patients, specifically Patients 3, 5, and 6, manifested pregnancy complications, including decreased fetal movement, low birth weight, and narrow hands. Postnatally, these individuals required special care and presented with sleeping problems, rapid weight gain, and genital abnormalities, which were observed particularly in Patients 2, 5, and 6. Notably, Patients 2, 3, and 6 (75%) exhibited hyperactivity/aggressiveness, and Patients 6 and 7 showed intellectual disability. Furthermore, Patients 2 and 6 (50%) underwent C‐sections, experienced seizures, hyperphagia, and fair skin, and demonstrated poor socialization. Patient 3 also displayed minor features such as dermatillomania, high pain tolerance, and colic.

The AS syndrome was diagnosed in two out of eight patients (25%), and intriguingly, both cases manifested almost all the cardinal phenotypes, including global developmental delay, poor socialization with cognition, seizures, happy demeanor, and fair skin (100%). Seizures were reported to be highly variable in severity and type, encompassing drop attacks, absence seizures, and nocturnal seizures, and were often difficult to control. Notably, Patient 7 exhibited additional features, such as pica, hyperactivity, aggressiveness, intellectual disability, colic, inattention, and impulsivity. In contrast, Patient 8 presented with distinct features, including facial dysmorphism, floppy baby syndrome, hypotonia, and sleeping problems. Both the PWS (3 out of 4) and AS (1 out of 2) individuals presented with sleeping problems, including issues such as apnea, decreased need for sleep, daytime sleepiness, or somnolence in their sleeping patterns.

Two out of the eight individuals (25%) included in this study were diagnosed with 15q duplication syndrome. These patients presented with mild global developmental delay and behavioral abnormalities such as poor socialization and cognition, and stereotypic behavior with autistic features. Patient 1 manifested floppy baby syndrome, thick viscous saliva, and a high pain threshold. Patient 4, on the other hand, exhibited rapid weight gain, stunted growth compared to familial standards, and infrequent episodes of vomiting.

### 3.5 Comparative analysis of the phenotypic consequences in eight patients

Our analysis revealed that global developmental delay was observed in all eight patients. In PWS, certain clinical features, such as pregnancy complications, decreased fetal movement, low birth weight, special care after birth, and floppiness, are only seen in the prenatal and neonatal periods. Moreover, PWS patients exhibit more prominent clinical phenotypes than the other two syndromes, including facial dysmorphisms, short stature, narrow hands, hypotonia, hyperactivity or aggressiveness, rapid weight gain, genital abnormalities, and sleeping problems. Although hyperphagia or polyphagia and rapid weight gain are usually seen in PWS cases, our cohort included one AS patient who showed a tendency toward pica, but their weight remained normal, and one patient with 15q11–q13 duplication syndrome who exhibited rapid weight gain. Patients with 15q11–q13 duplication syndrome exhibited some features such as repetition and restricted interests, as well as autism‐like phenotypes, which were not observed in the PWS and AS cases. In our study cohort, all the participants were under 6 years old and experienced speech delay; comprehensive psychological assessments or IQ tests were not conducted, hindering a definitive determination of intellectual disability or autism. Clinically, intellectual disability was suspected in Patients 6 and 7, whereas features indicative of autism were observed in Patients 1 and 4. Due to the age limitation and challenges posed by speech delay, these clinical suspicions should be carefully considered in the absence of formal psychological assessments. Seizures were observed in both PWS (two individuals) and AS cases, but AS patients tended to experience more severe seizures that were difficult to control and varied in type, including drop attacks, absence, and nocturnal seizures. Among all the cases studied in our cohort, PWS and AS cases exhibited more severe phenotypes than 15q duplication cases.

## 4 DISCUSSION

This study investigates a Bangladeshi NDD cohort, revealing that 3% (8/260) carry a pathogenic variant within the 15q11–q13 critical region. The chromosome 15q11–q13 critical region is a vital genetic locus that regulates several genes impacting neurodevelopment through genomic imprinting, including *UBE3A*. Despite arising from variations in the same genetic region, PWS, AS, and Dup15q syndrome are distinct NDDs that differ in their phenotypic manifestations and diagnostic strategies. Our study aims to determine the prevalence of deletion and duplication mutations in a cohort of eight Bangladeshi individuals suspected of having one of these three syndromes associated with the 15q11–q13 region. Notably, this study provides clinical insights from a genetically under‐representative population. The study cohort comprised individuals who displayed preliminary symptoms of PWS/AS, including developmental delay, behavioral abnormalities, facial dysmorphism, hypotonia, and rapid weight gain. Given that the characteristic signs of these disorders change with age and the initial symptoms overlap with those of other ailments, a clinical diagnosis of PWS/AS in early infancy can be challenging.

Previous studies have indicated that the median age of diagnosis in PWS patients ranged from 0.8 to 19.8 years, whereas in AS patients, it was 0.9 to 6.2 years (Faundes et al., 2015; Lo et al., 2012; Luk & Lo, 2016). Likewise, genetic screening was conducted in suspected individuals in the study cohort, and the median age of diagnosis was 1.7 years. Interestingly, a developmental delay was observed in all patients, constituting a 100% incidence rate in our study. Our findings are consistent with a previous report that has highlighted clinical manifestations in all three disorders (Kalsner & Chamberlain, 2015b). Chromosomal microarray analysis was performed as a first‐tier test on all eight patients and identified six distinct PWS/AS deletions and two duplications, each exhibiting different characteristics. Patient 1 displayed a significant 4.8‐megabase duplication spanning the BP1–BP3 locus (GRCh38 chr15:23123714‐28193120), affecting 15 imprinted genes and six non‐imprinted genes. In contrast, Patient 7 exhibited a 4.9‐megabase deletion characterized as a type 2 deletion, spanning the BP2–BP3 region (GRCh38 chr15:23374765‐28193120), removing approximately 15 imprinted genes and 6 non‐imprinted genes. Notably, the remaining subjects manifested infrequent and more extensive deletions or duplications that surpassed the BP1–BP3 locus (Smith & Hung, 2017; Urraca et al., 2013). These observations suggest significant heterogeneity in the 15q11–q13 chromosome region underlying PWS/AS/15q11–q13 duplication syndrome, which may contribute to the variable clinical presentations seen in affected individuals. Variable length gene that impacts the variable number of genes will have a difference in gene dosage and eventually will contribute to the phenotypic heterogeneity. To ascertain the accuracy of our CNV data obtained from microarray analysis, we utilized a highly sensitive method, ddPCR, to independently validate the CNV status of four samples. Interestingly, all four samples showed a 100% concordance rate between microarray and ddPCR results, significantly strengthening the reliability of our results. Collectively, our data provide robust evidence supporting the high accuracy and dependability of our CNV data.

The detection of copy numbers and methylation status in the critical region of 15q11.2–q13 is primarily accomplished through the utilization of MS‐MLPA. The assay demonstrates a high analytical sensitivity, surpassing 99% for PWS and 80% for AS detection. Widely adopted for distinguishing among deletion, UPD, and parental origin of duplications within the 15q11.2–q13 region, this assay elucidates the underlying mechanisms of three distinct disorders. Notably, it complements microarray findings, offering valuable insights into the etiology of 15q11.2–q13 rearrangements (Dawson et al., 2015). Given the economic challenges prevalent in Bangladesh, an economically underdeveloped country, the feasibility of conducting additional tests such as MS‐MLPA to unveil the methylation status and parental origin of the 15q11.2–q13 critical region becomes a pertinent consideration in the pursuit of comprehensive genetic investigations. Additionally, the patients underwent a comprehensive clinical examination, and based on their clinical phenotype and mutation type, they were classified into the three disorders (4 PWS, 2AS, and 2 15q_duplications (Figure 2). This finding aligns with the previous research indicating that CNVs in this region can lead to diverse clinical presentations. For instance, a study by Veltman et al. (2005) reported that individuals with PWS/AS deletions exhibit significant variability in cognitive function and behavior, even among those with the same deletion size (Buiting, 2010; Burnside et al., 2011; Milner et al., 2005). Moreover, the size and location of the CNVs did not always correspond with the severity of the clinical phenotype, adding to the complexity of these genetic disorders (Veltman & Brunner, 2012).

Our findings are consistent with the results of a Dutch cohort study involving 244 infants with PWS, which reported similar clinical phenotypes, such as reduced fetal movements in 78.5% of cases, hypotonia in all cases, and feeding difficulties, such as poor sucking and reliance on tube feeding, in 93.9% of cases (Grootjen et al., 2022). We also observed a range of behavioral issues, which are commonly seen in individuals with PWS. Significant hypotonia at birth and feeding difficulties can result in failure to thrive during infancy. This feeding difficulty typically progresses to hyperphagia in early childhood, which, if left uncontrolled, can lead to obesity and associated health complications (Gold et al., 2018). Further, as reported previously, two AS‐confirmed patients in our study exhibited seizures as a common phenotype along with behavioral abnormalities (Lossie et al., 2001; Shaaya et al., 2016). A meta‐analysis of CNVs in the 15q11–q13 region reported significant associations with ASDs, schizophrenia, and epilepsy (Moreira et al., 2014). In our study, individuals diagnosed with Dup15q syndrome have features of both PWS and AS, as well as some features unique to the disorder. Patients were observed to have some autistic features, but due to some constraints, psychological evaluations were not carried out to ensure autism (Piard et al., 2010).

Our study has yielded significant clinical insights, demonstrating considerable phenotypic heterogeneity both within and among these syndromes. Despite originating from genetic variations within the same region, these syndromes exhibit unique phenotypic characteristics. This underscores the distinct nature of each disorder and highlights the need for personalized diagnostic and therapeutic strategies that can effectively tackle the specific challenges posed by each syndrome.

## 5 CONCLUSION

We observed a total of 3.1% of Bangladeshi NDD cases impacted by 15q11–q13 critical region pathogenic variants. Our study has provided significant insights into the genotypic and phenotypic heterogeneity of NDDs linked to the chromosome 15q11–q13 region. The study has underscored the cruciality of prompt and precise diagnosis of PWS, AS, and Dup15q syndrome for effective clinical intervention and management, which can potentially benefit affected individuals and their families. Our study has emphasized the significance of merging genetic analysis with clinical observation to facilitate preliminary diagnosis and assist in the development of targeted clinical interventions and management strategies. These findings have great importance to the field of NDDs and will have consequential implications for future research in this area.

## AUTHOR CONTRIBUTIONS

**Rabeya Akter Mim**: Conceptualization; validation; formal analysis; writing—original draft; writing—review and editing; investigation; project administration. **Anjana Soorajkumar**: Conceptualization; methodology; validation; formal analysis; writing—original draft; writing—review and editing; investigation; project administration. **Noor Kosaji**: Methodology; formal analysis. **Muhammad Mizanur Rahman**: Methodology; data curation; investigation; validation; formal analysis. **Shaoli Sarker**: Methodology; data curation; investigation; validation; formal analysis. **Noushad Karuvantevida**: Methodology; formal analysis. **Tamannyat Binte Eshaque**: Methodology; data curation; investigation; validation; formal analysis; visualization. **Md Atikur Rahaman**: Methodology; data curation; investigation; validation; formal analysis; visualization; project administration; resources. **Amirul Islam**: Methodology; investigation; validation; formal analysis. **Mohammod Shah Jahan Chowdhury**: Methodology; data curation; investigation; validation; formal analysis; visualization; project administration; resources. **Nusrat Shams**: Methodology; data curation; investigation; validation; formal analysis; project administration; resources; visualization. **K.M. Furkan Uddin**: Data curation; investigation; validation; formal analysis; methodology; project administration; resources; visualization. **Hosneara Akter**: Conceptualization; methodology; supervision; project administration; writing—review and editing. **Mohammed Uddin**: Conceptualization; methodology; writing—review and editing; funding acquisition; supervision; investigation; validation; formal analysis; visualization; project administration; resources; writing—original draft; software.

## CONFLICT OF INTEREST STATEMENT

The authors declare no conflicts of interest.

### PEER REVIEW

The peer review history for this article is available at https://publons.com/publon/10.1002/brb3.3437.

## Supporting information

Figure S1 Summary of preliminary physical examination. The highlighted columns in the map display the patients with positive symptoms, whereas the gender of each patient is denoted by red letters (M for male and F for female). The bars represent the frequency of patients showing specific clinical features.

Supporting Information

Table S1 Molecular genetic study of eight patients in the Bangladeshi cohort
Table S2 ddPCR probes for validation
Table S3 PWS/AS breakpoints and genes

## Data Availability

Access to all data is available by contacting the corresponding author.
