# Supplementary material for: Expanding deep phenotypic spectrum associated with atypical pathogenic structural variations overlapping 15q11–q13 imprinting region
Source: Brain Behav. 2024 Apr 14;14(4):e3437. doi: 10.1002/brb3.3437 (PMC11016631; doi:10.1002/brb3.3437)
Supplement: Supplementary file 1 — Figure S1 Summary of preliminary physical examination. The highlighted columns in the map display the patients with positive symptoms, whereas the gender of each patient is denoted by red letters (M for male and F for female). The bars represent the frequency of patients showing specific clinical features. [file BRB3-14-e3437-s002.docx]

**Supplementary Figure**

**
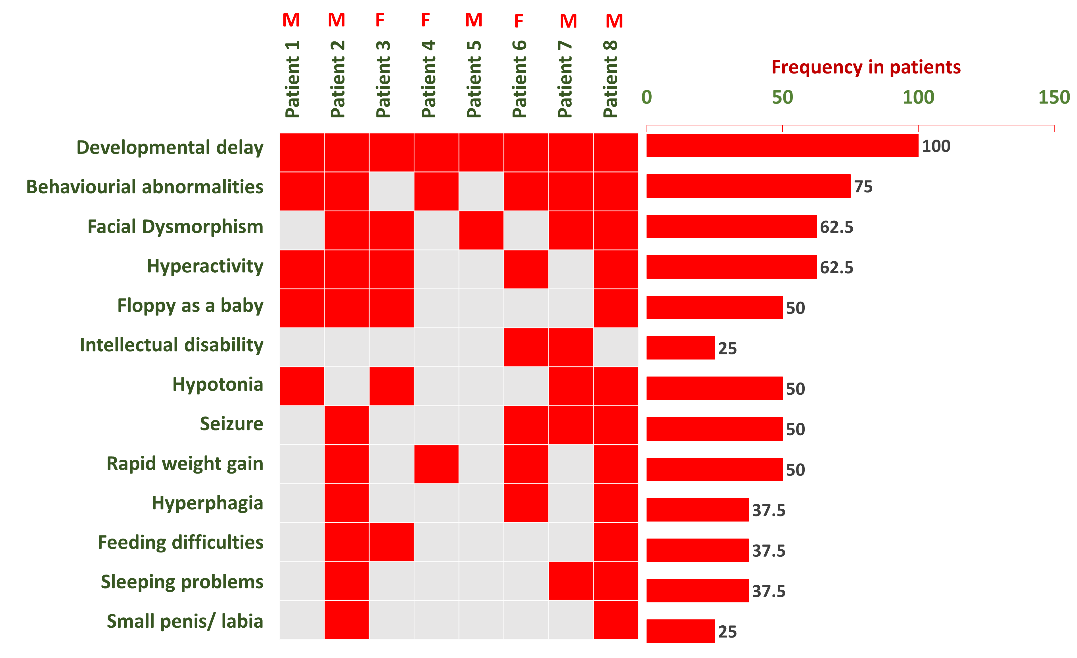
**

**Supplementary figure 1:** Summary of preliminary physical examination. The highlighted columns in the map display the patients with positive symptoms, while the gender of each patient is denoted by red letters (M for male and F for female). The bars represent the frequency of patients showing specific clinical features.
