# Supplementary material for: Expanding deep phenotypic spectrum associated with atypical pathogenic structural variations overlapping 15q11–q13 imprinting region
Source: Brain Behav. 2024 Apr 14;14(4):e3437. doi: 10.1002/brb3.3437 (PMC11016631; doi:10.1002/brb3.3437)
Supplement: Supplementary file 2 — Supporting Information [file BRB3-14-e3437-s003.docx]

**MATERIALS AND METHODS**

**Study Subjects and deep clinical assessment**

The study was conducted in accordance with ethical guidelines and received approval from the Institutional Review Board of Holy Family Red Crescent Medical College and Hospital in Dhaka, Bangladesh. The study cohort (N = 247) was derived from the NeuroGen Healthcare database located in Dhaka, Bangladesh. Thorough neurological and neurodevelopmental assessments were conducted by a team of experienced paediatric neurologists during clinical visits to identify potential individuals and establish clinical diagnoses of Prader-Willi syndrome (PWS) or Angelman syndrome (AS) based on the observed features. Prior to enrolment, individuals with suspected PWS/AS were invited to participate and provided written consent forms, which were approved by their parents or legal guardians. A comprehensive physical examination, including morphometric assessments, was performed to gather detailed data on the patients' physical characteristics. A total of eight patients were included in the study, and demographic and clinical information was obtained through formal parental interviews conducted during the sample collection at the clinic. This information encompassed baseline demographics such as age, gender, and birth history, as well as clinical details including developmental histories, medical histories, family history, infection histories, dietary details, and behavioral issues. All patients underwent in-depth clinical phenotyping, which involved multiple examination sessions to evaluate a wide range of clinical symptoms.

**Genomic DNA extraction**

Peripheral blood sample was collected (10 ml) from patients in an EDTA vacutainer tube. Genomic DNA was extracted using ReliaPrep™ Blood gDNA isolation kit (Promega, USA) following the protocols detailed in the kit. The quality and quantity of DNA were determined using NanoPhotometer C40 (Implen, Germany) and resolved on 0.8% agarose gel.

**Whole-genome microarray**

We conducted genome-wide microarray to identify chromosomal abnormalities such as deletions, duplications, translocations, and rearrangements. Changes in fluorescence intensity between the test specimen and the controls were investigated using array comparative genomic hybridization (aCGH) chip technology in the Agilent system. Digestion, ligation, PCR, labeling, hybridization, and scanning were all performed following standard protocols. This microarray uses 33,000 probes spread across the genome to detect 372 genetic abnormalities (including >60 loci in the DECIPHER database reported for neurodevelopmental disorders) and targets 41 subtelomeric regions that are vulnerable to chromosomal abnormalities. We rigorously applied multiple algorithmic techniques (MATLAB and Java) and manual curated the data to pinpoint genomic variation based on the normalized log2 intensities of the probes. A circular binary segmentation algorithm was used on the normalized –log2 values to detect copy number variations (CNVs). Horizon platform from GenomeArc Inc. was used for clinical annotations of the SVs and classifies pathogenicity following American College of Medical Genetic (ACMG) guidelines. Horizon algorithm was integrated with in house control population (9610 samples) to exclude common CNVs and retain only rare CNVs for clinical annotation. We also integrated the Horizon platform with previously published neurodevelopmental cohorts to identify variant frequencies in disease cohorts [Akter et al., 2023; Uddin et al., 2016].

**NDD Disorder Enrichment**

We have screened all known disorders from DECIPHER database and identified variant enrichment within this cohort. CNVs can vary in actual length compared to the breakpoints mentioned in disorder database. Applying Horizon analysis platform, we have conducted enrichment analysis by reciprocal mapping the CNV breakpoints, where at least > 60% of the variant must overlap between a CNV and a disorder breakpoint. Our analysis demonstrates, 15q11-q13 imprinting region shows the most enriched number of NDD cases within the cohort. We have proceeded for all related downstream analysis (variant validation, appointment for deep phenotype information) based on the cases that are within the 15q11-q13 imprinting region.

**Droplet digital PCR**

Copy number assays were performed using the Droplet Digital PCR (ddPCR) System (Bio-Rad Laboratories, Inc.). The GeneAssist™ Copy Number Assay Workflow Builder (Thermo Fischer, United States) was used to design TaqMan assays on Chr15, SNHG14 (Hs05375107_cn) with FAM dye. TaqMan™ Copy Number Reference Assay, human, RNase P with VIC dye was used as a reference assay. A total of 22 µL reaction mix was prepared, containing 3.5 µL of template DNA (20 ng/μL) without restriction digestion, 10 µL of 2X ddPCR supermix for probes (no UDP) (Bio-Rad Laboratories Inc.), 1 µL each 20X TaqMan target probe (FAM) and 20X TaqMan reference probe (VIC) (Applied Biosystems, United States), and 6.5 µL of RNase-/DNase-free water. All reactions were prepared in triplicates with one negative control. The reaction mixtures were partitioned using the QX200 Droplet Generator™ and then transferred to a 96-well plate and amplified using the C1000 Touch thermal cycler, following the manufacturer's protocol. The samples were then read using the QX200 Droplet Reader™. Data acquisition and analysis were performed using QuantaSoft Version 1.7.4.0917, and the Poisson algorithm was used to determine the concentrations of the targets as copies/μl.
